# An Approach to Establishing Diagnostic Reference Levels in Interventional Pediatric Cardiology from Different Regions of Brazil

**DOI:** 10.3390/children11020200

**Published:** 2024-02-05

**Authors:** Viviane K. Asfora, Marcelo B. Freitas, Regina B. Medeiros, Hugo R. Schelin, Akemi Yagui, Marcus V. L. Oliveira, Luiz M. S. A. Leite, Guillermo A. Lopez, Maryanna R. S. Roberto, Fabio L. Gagetti, Pablo H. O. Souza, Bruna Vargas, Valeriy V. Denyak, Helen J. Khoury

**Affiliations:** 1 Departamento de Energia Nuclear, Universidade Federal de Pernambuco, Recife 50740-540, Brazil; viviane.asfora@ufpe.br (V.K.A.); luiz.aguiarleite@ufpe.br (L.M.S.A.L.); maryanna.regina@ufpe.br (M.R.S.R.); helen.khoury@ufpe.br (H.J.K.); 2 Departamento de Biofísica, Escola Paulista de Medicina, Universidade Federal de São Paulo, São Paulo 04023-062, Brazil; 3 Programa de Pós-Graduação em Medicina (Cardiologia), Universidade Federal de São Paulo, São Paulo 04024-002, Brazil; medeiros@unifesp.br; 4 Instituto de Pesquisa Pelé Pequeno Príncipe, Faculdades Pequeno Príncipe, Curitiba 80250-209, Brazil; hugo.schelin@professor.fpp.edu.br (H.R.S.); akemi.yagui@aluno.fpp.edu.br (A.Y.); bruna.vargas@aluno.fpp.edu.br (B.V.); 5 Departamento de Tecnologia em Saúde e Biologia, Instituto Federal da Bahia, Salvador 40301-015, Brazil; marcusoliveira@ifba.edu.br (M.V.L.O.); guillermolopez@ifba.edu.br (G.A.L.); 6 Programa de Residência em Física Médica, Universidade Federal de São Paulo, São Paulo 04021-001, Brazil; fabio.gagetti@unifesp.br (F.L.G.); henrique.pablo@unifesp.br (P.H.O.S.); 7 Programa de Pós-Graduação em Engenharia Biomédica, Universidade Tecnológica Federal do Paraná, Curitiba 80230-901, Brazil; denyak@utfpr.edu.br

**Keywords:** pediatric cardiology, fluoroscopy-guided interventional radiology, radiation dose, air kerma–area product, diagnostic reference level

## Abstract

Diagnostic reference levels (DRLs) are a pivotal strategy to be implemented since pediatric interventional cardiology procedures are increasing. This work aimed to propose an initial set of Brazilian DRLs for pediatric interventional diagnostic and therapeutic (D&T) procedures. A retrospective study was carried out in four Brazilian states, distributed across the three regions of the country. Data were collected from pediatric patients undergoing cardiac interventional procedures (CIPs), including their age and anthropometric characteristics, and at least four parameters (number of images, exposure time, air kerma–area product—*P*_KA_, and cumulative air kerma). Data from 279 patients undergoing CIPs were gathered (147 diagnostic and 132 therapeutic procedures). There were no significant differences in exposure time and the number of images between the D&T procedures. A wide range of *P*_KA_ was observed when the therapeutic procedures were compared to diagnostics for all age groups. There were significant differences between the D&T procedures, whether grouping data by patient weight or age. In terms of cumulative air kerma, it was noted that no value exceeded the level to trigger a monitoring process for patients. This study shows that it is possible to adopt them as the first proposal to establish national DRLs considering pediatric patient groups.

## 1. Introduction

Radiation exposure must be monitored in pediatric interventional cardiology procedures for congenital heart diseases, given the vulnerability of children to the potential risks of stochastic and deterministic effects in these procedures that may involve high doses. These complex procedures require well-trained specialists, knowledgeable in technology and radiation protection, to decide on technical strategies able to minimize exposure. Specialists must be aware of potential long-term radiation effects when determining treatment strategies for this exposed population [1,2].

In 2022, Brazil had an estimated total population of around 213 million people, of whom about 60 million were children aged 0 to 16 years [3]. Data from 2020 indicate 2762 live births with congenital heart diseases. In a study conducted by Soares et al. [4], it was found that over 50% of children with congenital heart diseases had a low birth weight, which increases the difficulty of treatment. Despite their complexity, these procedures are increasing in Brazil, with more than 50% of the states now having pediatric hemodynamics units.

A recent publication by the International Atomic Energy Agency (IAEA) emphasized the benefits of dose monitoring to optimize radiological protection for individual patients and the general population. Manual patient dosimetry surveys have been used for many years, and currently, data are often collected automatically using digital imaging systems [5]. Automated systems have the advantage of providing robust statistical data for the estimation of diagnostic reference levels (DRLs), allowing dose optimization strategies to be implemented.

Reports from modern fluoroscopic systems provide at least four parameters at the end of the procedure: fluoroscopy time, the number of fluoroscopic images, air kerma–area product (*P*_KA_), and cumulative air kerma at the patient entrance reference point (*K*_a,r_) [6,7,8].

*P*_KA_ is a reasonable indicator of the risk of stochastic effects, and *K*_a,r_ is a useful predictor of skin dose and, therefore, radiation-induced skin injury. Cumulative fluoroscopy time can be assessed at the end of the procedure but has been shown to poorly correlate with the peak skin dose.

In the USA, *K*_a,r_ is more widely available because it is required by the US Food and Drug Administration for all fluoroscopic equipment, but *P*_KA_ is not required to be displayed. In Europe, *P*_KA_ is more widely used [9]. For purposes of comparison with DRLs, both quantities are acceptable [8].

If the median *P*_KA_ and/or *K*_a,r_ in a particular institution exceeds the corresponding DRL value, evaluation of fluoroscopy time and the number of images acquired may help to determine whether these are contributing factors. They may help to clarify the reasons, suggesting the need for optimization [9].

General pediatrics has unique features related to the wide range of patient sizes. These ranges are based on child growth standards and are used to estimate DRLs for different age and weight ranges [9].

The analysis of these patient dose metrics and the establishment of diagnostic reference levels (DRLs), classified by weight and age group, can help formulate a strategy to optimize doses and avoid unnecessarily high doses in pediatric patients [10]. Motivated by the participation in the “Optimization of Protection in Pediatric Interventional Radiology in Latin America and the Caribbean” (OPRIPALC) initiative launched in 2018 [11,12], this study aims to propose a first set of Brazilian DRLs for pediatric interventional diagnostic and therapeutic procedures. The study also aims to identify potential differences in medical practices between the different regions of Brazil.

## 2. Materials and Methods

This retrospective study covered four Brazilian states in three regions of the country (Table 1): the southeast region (the city of São Paulo, state of São Paulo), the south region (the city of Curitiba, state of Paraná), and the northeast region (the city of Recife, state of Pernambuco, and the city of Salvador, state of Bahia). All participating institutions are members of the OPRIPALC initiative. The data presented in this paper were collected from April 2019 to June 2023.

Data were collected on pediatric patients undergoing cardiac interventional procedures, both diagnostic and therapeutic, including their age and anthropometric characteristics, limited to body weights up to 80 kg, according to the ICRP [9]. Patients were respectively grouped according to age and weight groups: <1 year, 1 to <5 years, 5 to <10 years, 10 to <15 years, and >15 years and <5 kg, 5–15 kg, 15–30 kg, 30–50 kg, and 50–80 kg. The type of procedure performed (either diagnostic or therapeutic) and its technical characteristics, as well as the *P*_KA_, *K*_a,r_, exposure time to X-rays (fluoro/cine), and the total number of images (image acquisition runs), were recorded at the end of each procedure, except for the number of images in the D facility.

Data were organized by weight and age groups to determine national typical values using statistical variables of the distributions (minimum, mean, median, 75th percentile or 3rd quartile, and maximum) as recommended by the ICRP [9]. Manual data collection was used because of the lack of an automated dose monitoring system in the participating hospitals. All data were anonymized, collected by a regional member and processed by the Brazilian members of OPRIPALC. The study adhered to the Declaration of Helsinki, and the protocol was approved by the Brazilian National Research Ethics System (CONEP) (CAAE 59399222.2.0000.9030 and 08746819.0.0000.5580).

All angiographic devices underwent annual quality control tests based on Brazilian national regulations to ensure that they were in suitable condition for use in healthcare settings [13,14]. Table 1 shows some of the characteristics of the angiographic devices installed in the four different regions of Brazil.

The hospital in São Paulo (Institution A) is a general medical center with highly complex healthcare services and, as a university hospital, is responsible for professional training. Curitiba Hospital (Institution B) is a center specialized in pediatric care, considered a reference hospital, and also has a research institute. Recife Hospital (Institution C) is a general care center of high regional relevance. Salvador Hospital (Institution D) is a national reference hospital with forty medical specialties, including cardiology and pediatrics.

## 3. Results

### 3.1. Patient Characteristics

Data were collected from 279 patients undergoing cardiac interventional procedures, of whom 147 underwent diagnostic procedures and 132 underwent therapeutic procedures. The percentage of male patients undergoing diagnostic procedures was 53%, while the percentage of female patients was 47%. For therapeutic procedures, 69% of patients were female and 31% were male. Table 2 and Table 3 present the descriptive statistics of the anthropometric characteristics of patients undergoing therapeutic and diagnostic procedures, respectively, classified by age and weight groups.

### 3.2. Irradiation Parameters

**Figure 1:**
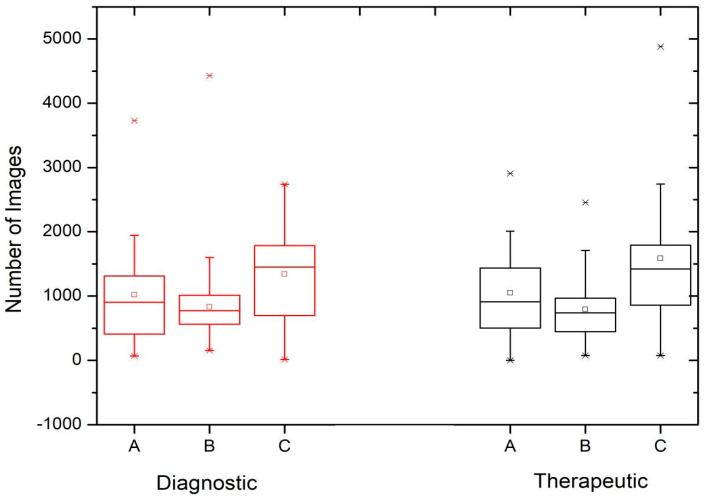
Number of images captured during diagnostic (D) and therapeutic (T) procedures at the hospitals participating in the survey: São Paulo (**A**) (n_D_ = 31 and n_T_ = 18), Curitiba (**B**) (n_D_ = 79 and n_T_ = 52), and Recife (**C**) (n_D_ = 16 and n_T_ = 44).

**Figure 2:**
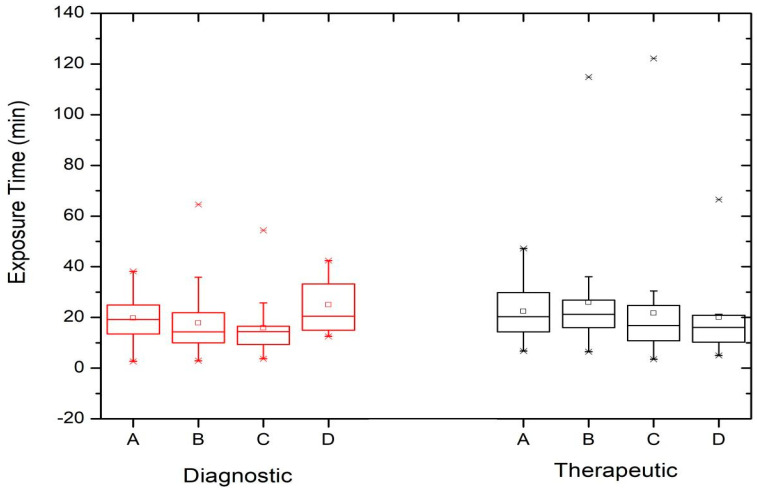
Exposure time for diagnostic (D) and therapeutic (T) procedures performed at the institutions involved in this study: São Paulo (**A**) (n_D_ = 31 and n_T_ = 18), Curitiba (**B**) (n_D_ = 79 and n_T_ = 52), Recife (**C**) (n_D_ = 16 and n_T_ = 44), and Salvador (**D**) (n_D_ = 21 and n_T_ = 18).

Figure 1 shows the image acquisition runs (number of images) during diagnostic and therapeutic procedures performed at three out of the four centers (São Paulo, Curitiba, and Recife as Institution A, B, and C, respectively). The data are presented using a box and whisker plot, where the box plot shows the first and third quartiles, together with the maximum and minimum values (the horizontal bar indicates the median). Outliers and mean values are indicated respectively by the line cross and the open square. The number of diagnostic (n_D_) and therapeutic (n_T_) procedures performed at each institution and contributing to each box and whisker plot is also shown. Figure 2 shows the variation in exposure time for both diagnostic and therapeutic procedures at the four institutions evaluated in this study, including the hospital located in the city of Salvador (Institution D).

Figure 1 shows that the results of the image acquisition runs (number of images) are very similar when comparing the diagnostic and therapeutic procedures. Differences in the third quartile values of the exposure time distributions between the diagnostic and therapeutic procedures can be observed in Figure 2, suggesting that, with the exception of Institution D, therapeutic procedures generally require higher exposure times, which can be explained by the complexity of pathologies and procedures. These differences become more apparent when considering procedures with longer exposure times.

### 3.3. Patient Doses

**Figure 3:**
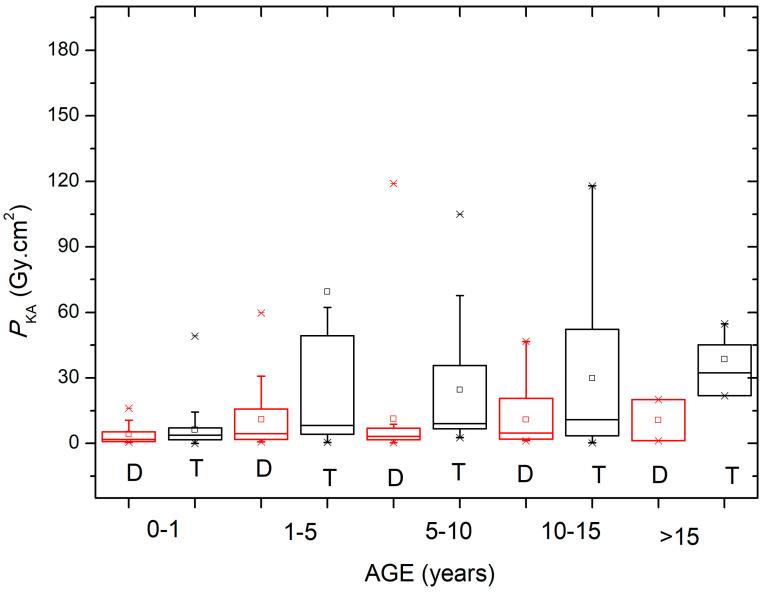
*P*_KA_ values obtained during the diagnostic (D) and therapeutic (T) procedures for the different patient age groups.

**Figure 4:**
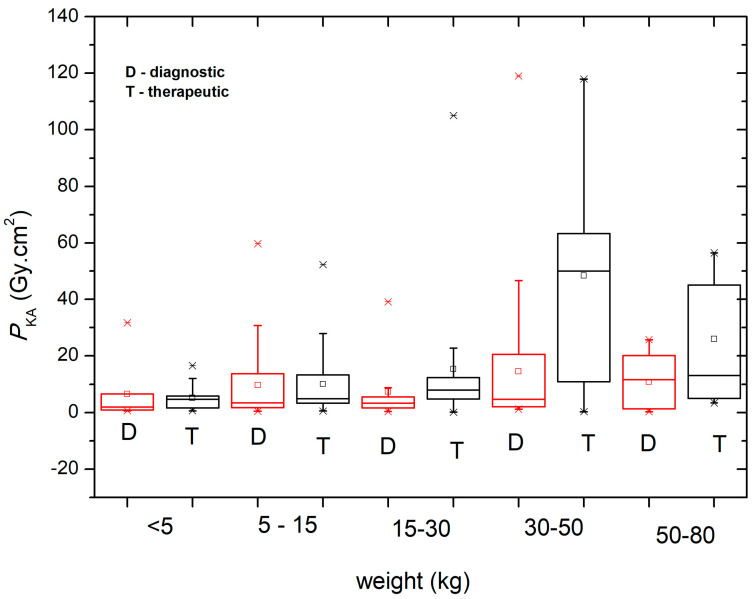
*P*_KA_ values obtained during the diagnostic (D) and therapeutic (T) procedures for the different patient weight groups.

Figure 3 shows the *P*_KA_ values obtained during the diagnostic (D) and therapeutic (T) procedures in the different pediatric age groups at the four participating centers. Figure 4 shows the *P*_KA_ values obtained during the diagnostic (D) and therapeutic (T) procedures categorized by the weight groups of the pediatric patients.

A box and whisker representation was chosen to present the data, with the box plots showing the first and third quartiles, along with the maximum and minimum values (the horizontal bar represents the median). Outliers and mean values are indicated respectively by the line cross and the open square. The values can be more easily compared using Table 4, which shows the median and third quartile of *P*_KA_ for the diagnostic and therapeutic procedures based on the patient age and weight groups. It can be seen that the median and third quartile values are higher for therapeutic procedures when compared to diagnostic procedures.

**Figure 5:**
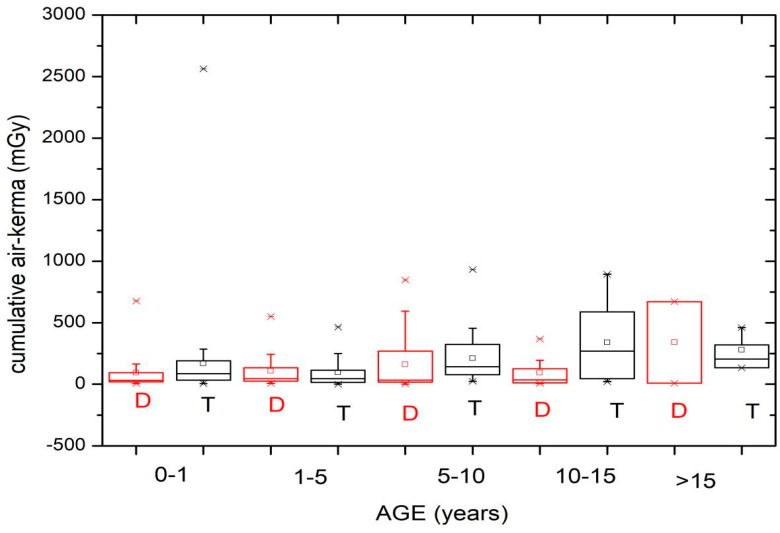
*K*_a,r_ values obtained during the diagnostic (D) and therapeutic (T) procedures for the different patient age groups.

**Figure 6:**
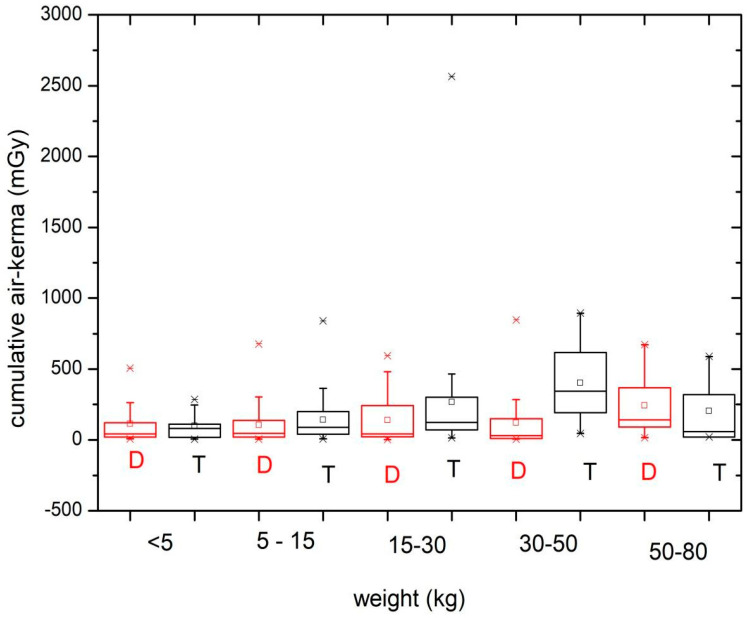
*K*_a,r_ values obtained during the diagnostic (D) and therapeutic (T) procedures for the different patient weight groups.

Figure 5 and Figure 6 show the cumulative air kerma values obtained during the diagnostic (D) and therapeutic (T) procedures according to the patient age and weight groups. Table 5 shows the median and third quartile of *K*_a,r_ for the diagnostic and therapeutic procedures, taking into account the patient age and weight groups.

The values can be compared more easily with Table 5, which shows the median and third quartile of *K*_a,r_ (mGy) for the diagnostic and therapeutic procedures based on the patient age and weight groups. It can be seen that, with some exceptions, the median and third quartile values for therapeutic procedures are higher than for diagnostic procedures.

The values for the air kerma–area product (Table 4) and cumulative air kerma (Table 5) for therapeutic procedures increase with weight and age group, except for the last group.

## 4. Discussion

For pediatric interventional cardiology procedures, there are currently no dose reference levels (DRLs) established based on national dose surveys in Brazil. This study presents a first dataset and highlights the need for further refinement, taking into account the geographical dimensions of the country and the specificities associated with cardiac interventional procedures.

There is a consensus that the implementation of DRLs in pediatric interventional cardiology procedures is not as straightforward as it might be for other imaging modalities. It is possible to identify several difficulties that result from the wide distribution of DRLs, such as the complexity of diseases and clinical/therapeutic procedures, and the characteristics of the facilities, such as technology, staff expertise, equipment performance, and specific protocols considering image quality, among others.

Therapeutic procedures are diverse and less standardized than diagnostic procedures. In addition, they may be performed immediately after a diagnostic procedure. This partly explains why they have higher variability in dosimetric indicator values.

Complex interventional procedures have been shown to result in high skin doses in adults and high absorbed doses in exposed organs and tissues in children. The potential clinical effects of single-delivery radiation doses to the skin in adults have been studied, but no data are available for children yet [2].

In this study, the sample composition included facilities with different characteristics, such as private centers, public centers, and dedicated pediatric health centers, as recommended by the European Guidelines on DRLs for Paediatric Imaging, 2015 [15]. Due to the significant differences in the complexity of the procedures, it was challenging to ensure a minimum of 20 patients per type of procedure for each group and facility, as desired. Consequently, it was not possible to compare outcomes between centers. This study is ongoing, and it is intended to expand the data collection to allow quantitative data collection to statistically assess variations in radiation exposure.

A minimum of 15 patients (30–50 kg group) and up to 67 patients (5–15 kg group) were collected to estimate national DRLs. Given the sample size, the results can be considered as a first set of national DRLs for diagnostic and therapeutic procedures in interventional pediatric cardiology.

The characteristics of the patients by age and weight groups were very similar between diagnostic and therapeutic procedures (Table 2 and Table 3), although the weight of patients undergoing therapeutic procedures was slightly lower than those undergoing diagnostic procedures.

There were no significant differences in exposure time and the number of images (frames) between the diagnostic and therapeutic procedures (Figure 1 and Figure 2). A wide range in the number of images was observed, indicating the variable complexity of the procedures and/or the lack of standardized protocols for procedures in the same center. Image acquisition runs should only be performed when necessary for diagnosis or the assessment of outcomes after a procedure.

It is noticeable that the number of images at center B is lower than at A and C, which could be related to the protocol chosen, reinforced by the fact that in this service, the procedures were performed by the same specialist. At center C, the number of images was higher than in the other centers, which could be explained by the fact that this service took place in a general care center, where the documentation of procedures may be relevant to justify the diagnosis or treatment of patients. Procedures with high exposure times (outliers), mainly in therapeutic ones, will be investigated to justify or identify the factors that led to these values. Based on the results of this study, optimization actions should be implemented at each center.

The lowest number of frames per second required to achieve the clinical objective should be used, and images should be obtained by using the lowest magnification (post-processing magnification is possible) [2].

In general, a wide range in *P*_KA_ was observed when comparing the therapeutic and diagnostic procedures for all age groups (Figure 3). When *P*_KA_ data were grouped according to patient weight, a large variation was observed in the range of 30–50 kg for therapeutic procedures (Figure 4). The *P*_KA_ values (median and third quartile) shown in Table 4 reinforce the findings of similar studies, pointing out that there are significant differences between diagnostic and therapeutic procedures, whether data are grouped by patient weight or age.

The median *P*_KA_ values found in this study were in most cases lower than those published in other studies (Table 6 and Table 7), when diagnostic procedures were considered, grouped by patient weight range. In particular, when compared with another large-scale study carried out in Brazil [16], the results of this study are always lower, whether for diagnostic or therapeutic procedures, grouped by patient age or weight. This finding can be explained by the fact that the institutions taking part in this study only use technology with flat-panel detector systems, in which the doses required to produce quality images are lower. Taking into account the therapeutic procedures, the results showed more variability, which requires a more detailed study considering the type of therapeutic procedure performed. In this case, attention should be paid to variations in pathology, technical parameters tailored to pediatric patients (protocol), among others.

When analyzing the cumulative air kerma values (Figure 5 and Figure 6), it is noted that only 1 value exceeded 2 Gy, pointing out that no value exceeded the trigger level (3 Gy for adult patients) to implement the clinical monitoring of potential harmful radiation injuries to the skin [8].

## 5. Conclusions

This study presents the first set of national diagnostic reference levels (DRLs) for pediatric interventional diagnostic and therapeutic procedures, based on the third quartile of *P*_KA_ distributions categorized by age and weight groups.

The results, which are based on three regions of the country and fall within the scope of other international initiatives, suggest that they can be considered as being a first proposal for the establishment of national diagnostic reference levels (DRLs) taking into account patient groups.

Significant differences in *P*_KA_ were found between the diagnostic and therapeutic procedures, taking into account patient groups (age and weight). Cumulative air kerma data were below the injury threshold in all but one patient, highlighting the importance of implementing DRLs to assist in the optimization process.

Detection systems with flat panel technology used in all institutions participating in this study may explain the lower values of the air kerma–area product found in this study.

Optimization strategies for diagnostic and therapeutic procedures should be implemented, focusing on the identified discrepancies (outliers) of dose indicators (air kerma–area product and cumulative air kerma) and exposure parameters (exposure time and number of images) determined at the different participating centers.

The participation of the institutions in this study, through the OPRIPALC program, facilitated the exchange of experiences among different centers in Brazil and Latin America, laying the groundwork for the implementation of this survey at the national level.

## Figures and Tables

**Table 1:** Location of the facilities and characteristics of the angiographic equipment used for pediatric procedures.

Location		Equipment Characteristics		
City Geographic Region	Type (Identification)	Manufacturer ^1^	Model	Detector System Technology
São Paulo Southeast	Public (Institution A)	Philips	Allura Xper FD20	Flat panel
		GE	Innova 3100	Flat panel
Curitiba South	Philanthropic (Institution B)	GE	Innova IGS 530	Flat panel
Recife Northeast	Private (Institution C)	Philips	Azurion 7	Flat Panel
Salvador Northeast	Philanthropic (Institution D)	Siemens	Artis Zee	Flat panel

^1^ Philips: Philips Medical System, Amsterdam, Netherlands; GE: General Electric Company, Massachusetts, USA; Siemens: Siemens Healthcare, Erlangen, Germany.

**Table 2:** Characteristics of the age groups of patients (years) undergoing therapeutic and diagnostic procedures.

Age Groups (Years)	Procedures ^1^	n ^2^	Mean ± SD ^3^	Median (IQR ^4^)	Min–Max
<1	D	36	0.39 ± 0.34	0.30 (0.10–0.58)	0.02–1.00
	T	48	0.40 ± 0.36	0.33 (0.04–0.75)	0.01–1.00
1–5	D	57	2.8 ± 1.1	2.5 (1.75–3.4)	1.1–5.0
	T	37	2.7 ± 1.0	3.0 (2.0–3.3)	1.1–5.0
5–10	D	29	7.1 ± 1.4	6.9 (6.0–8.0)	5.3–10.0
	T	22	6.9 ± 1.4	7.0 (5.1–8.3)	5.0–9.5
10–15	D	19	12.2 ± 1.2	12.1 (11.0–12.9)	10.0–14.7
	T	21	11.5 ± 1.3	11.0 (10.1–12.1)	10.0–14.5
>15	D	6	16.7 ± 0.2	16.6 (16.5–16.7)	16.3–17.0
	T	4	16.1 ± 0.9	16.1 (15.3–16.9)	15.4–17.1

^1^ D: diagnostic; T: therapeutic; ^2^ n: sample size; ^3^ SD: standard deviation; ^4^ IQR: interquartile (1st quartile–3rd quartile).

**Table 3:** Characteristics of the weight groups of patients (kg) undergoing therapeutic and diagnostic procedures.

Weight Groups (Kg)	Procedures ^1^	n ^2^	Mean ± SD ^3^	Median (IQR ^4^)	Min–Max
<5	D	17	4.5 ± 0.3	4.4 (3.7–4.2)	2.0–5.0
	T	24	3.6 ± 0.9	3.6 (3.1–4.0)	1.8–5.0
5–15	D	67	10.4 ± 2.5	10.6 (6.7–12.0)	5.2–15.0
	T	44	9.7 ± 2.7	10.0 (7.0–13.0)	5.1–14.5
15–30	D	27	20.6 ± 2.9	20.0 (18.4–23.2)	16.0–26.6
	T	34	20.0 ± 4.3	20.0 (16.0–23.0)	15.0–29.0
30–50	D	21	42.6 ± 4.1	41.9 (36.0–44.0)	31.0–50.0
	T	15	38.5 ± 6.8	37.3 (32.0–45.5)	31.0–51.0
50–80	D	7	62.2 ± 10.0	64.0 (52.0–69.0)	52.0–80.0
	T	8	62.8 ± 9.9	63.8 (51.7–71.0)	51.0–80.0

^1^ D: diagnostic; T: therapeutic; ^2^ n: sample size; ^3^ SD: standard deviation; ^4^ IQR: interquartile (1st quartile–3rd quartile).

**Table 4:** Median and third quartile of *P*_KA_ (Gy·cm^2^) in accordance with the weight and age groups of the patients for diagnostic and therapeutic procedures.

Air Kerma–Area Product (Gy·cm^2^)				
Procedure	Diagnostic		Therapeutic	
Weight groups (kg)	Median	3rd quartile	Median	3rd quartile
<5	1.5	4.1	4.7	5.9
5–15	3.4	13.7	4.9	13.3
15–30	3.3	5.4	8.1	15.3
30–50	5.1	20.6	52.7	63.3
50–80	10.8	12.2	17.4	49.9
Age groups (years)	Median	3rd quartile	Median	3rd quartile
<1	1.9	6.6	3.8	7.7
1–5	4.5	16.2	8.3	48.2
5–10	3.3	8.5	9.0	35.7
10–15	5.4	16.4	11.1	55.4
>15	10.7	-	38.7	49.9

**Table 5:** Median and third quartile of *K*_a,r_ (mGy) in accordance with the weight and age groups of the patients for diagnostic and therapeutic procedures.

Cumulative Air Kerma (mGy)				
Procedure	Diagnostic		Therapeutic	
Weight groups (kg)	Median	3rd quartile	Median	3rd quartile
<5	43.2	121.0	79.6	110.0
5–15	47.7	137.2	91.4	207.1
15–30	42.2	244.0	124.0	270.0
30–50	36.8	171.9	361.0	601.6
50–80	141.0	345.8	96.6	354.5
Age groups (years)	Median	3rd quartile	Median	3rd quartile
<1	31.1	95.0	86.9	186.5
1–5	47.4	139.0	45.4	112.0
5–10	41.8	273.5	142.6	319.4
10–15	45.3	119.8	284.0	596.7
>15	340.7	506.6	261.8	354.5

**Table 6:** Comparison of median and third quartile *P*_KA_ values (Gy·cm^2^) for interventional pediatric cardiology (diagnostic and therapeutic) reported in this and other papers according to patient age groups.

Age Groups (years)	*P*_KA_ (Gy·cm^2^)	Ubeda et al., 2012 [17]	Ubeda et al., 2015 [18]	Manica et al., 2020 [16]	This Paper	Ubeda et al., 2012 [17]	Ubeda et al., 2015 [18]	Manica et al., 2020 [16]	This Paper
		Diagnostic				Therapeutic			
<1	Median	1.0	-	3.0	1.9	0.9	-	2.5	3.8
	3rd quartile	-	1.17	7.5	6.6	-	1.11	5.7	7.7
1–5	Median	1.5	-	5.2	4.5	1.4	-	6.0	8.3
	3rd quartile	-	1.74	10.4	16.2	-	1.9	13.3	48.2
5–10	Median	2.2	-	13.4	3.3	1.9	-	11.9	9.0
	3rd quartile	-	2.83	21.8	8.5	-	3.2	21.3	35.7
10–15	Median	7.9	-	17.4	5.4	4.5	-	27.7	11.1
	3rd quartile	-	7.34	45.2	16.4	-	8.7	84.0	55.4
>15	Median	-	-	21.8	10.7	-	-	117.2	38.7
	3rd quartile	-	-	37.4	-	-	-	283.6	49.9

**Table 7:** Comparison of median and third quartile *P*_KA_ values (Gy·cm^2^) for interventional pediatric cardiology (diagnostic and therapeutic) reported in this and other papers according to patient weight groups.

Weight Groups (kg)	*P*_KA_ (Gy·cm^2^)	Ubeda et al., 2012 [17]	Ubeda et al., 2015 [18]	Manica et al., 2020 [16]	This Paper	Ubeda et al., 2012 [17]	Ubeda et al., 2015 [18]	Manica et al., 2020 [16]	This Paper
		Diagnostic				Therapeutic			
<5	Median	0.25	-	1.4	1.5	0.23	-	1.4	4.7
	3rd quartile	0.41	4.3	2.8	4.1	0.43	5.2	3.5	5.9
5–15	Median	1.0	-	5.7	3.4	0.93	-	5.7	4.9
	3rd quartile	1.6	5.0	11.1	13.7	1.7	8.0	14.0	13.3
15–30	Median	2.3	-	12.8	3.3	2.1	-	12.8	8.1
	3rd quartile	3.7	12.6	24.9	5.4	3.8	15.6	31.4	15.3
30–50	Median	4.0	-	22.8	5.1	3.7	-	22.8	52.7
	3rd quartile	6.5	43.4	44.2	20.6	6.8	25.5	55.8	63.3
50–80	Median	6.5	-	37.1	10.8	6.0	-	37.1	17.4
	3rd quartile	10.6	31.3	71.8	12.2	11.0	30.2	90.7	49.9

## Data Availability

The data presented in this study are available on request from the corresponding author. The data are not publicly available due to ethical and privacy restrictions.
